# Why is it so difficult to understand why we don’t understand human systemic lupus erythematosus? Contemplating facts, conflicts, and impact of “the causality cascade paradigm”

**DOI:** 10.3389/fimmu.2024.1507792

**Published:** 2025-01-28

**Authors:** Ole Petter Rekvig

**Affiliations:** ^1^ Fürst Medical Laboratory, Oslo, Norway; ^2^ Department of Medical Biology, Faculty of Health Sciences, UiT The Arctic University of Norway, Tromsø, Norway

**Keywords:** systemic lupus erythematosus (SLE), SLE classification criteria, the causality principle, the causality cascade paradigm, dynamically changing DNA structures, lupus nephritis pathogenesis

## Abstract

In attempts to understand systemic lupus erythematosus (SLE), we find ourselves in the intellectual cross-point between nosology, pathogenicity-oriented science, philosophy, empiricism, and qualified conjectures. A vital consequence in science theory is that scientific hypotheses that are not critically investigated are in danger of being transformed into scientific dogmas^1^. This statement has consequences for this study. Two central problematic aspects are discussed. For the first, we have to consider new selection principles for classification criteria—implying integration of the causality principle. Second, central historical data must be implemented if we aim to understand SLE. These data comprise famous descriptions of distinct, dynamically changing DNA structures linked to the genetic machinery. These unique structures have since their discoveries decades ago mostly been ignored in SLE research. Likewise, inconclusive dogmatic data indicate that different glomerular ligands are recognized by nephritogenic anti-dsDNA antibodies—exposed chromatin fragments or inherent membrane ligands. These incongruent models have not been comparatively and systematically investigated. Three research areas will be critically discussed: (*i*) selection and role of SLE classification criteria, a process that must imply the causality principle; (*ii*) definition and impact of anti-dsDNA structure-specific antibodies; (*iii*) incongruent pathogenic models that account for lupus nephritis. A precise and critically important question is if SLE itself is a response to a dominant unified cause that initiates a cascade of downstream effects (criteria) or if SLE represents combined responses to a random interplay of multiple cause-effect events. These principally different explanations are formally not excluded or accepted today. Currently, SLE may be regarded as a disease with phenotypic diversity, independently segregated manifestations with unresolved etiologies that are not unique to a single SLE phenotype. The focus for the present discussion is basically how we, by critical hypotheses, can re-consider science-based selection of SLE classification criteria in order to delimitate and rationalize SLE. Classification criteria, autoimmunity, DNA structures, and anti-dsDNA antibodies are integrated aspects in this discussion.

## A personal account and a provocative challenge

***“Ask critical questions provided they are based on reflections and science-based hypotheses, not on opportunistic assumptions or popular trends. Critical questions alter science” Hans Tuppy 2009***

Hans Tuppy^2^, professor in Biochemistry at the University of Vienna, died 24 April 2024. He was my first mentor when I studied Medicine at the University of Vienna. Through my interest in Biochemistry, we developed a communicative relationship: he encouraged me to become a scientist. The last time I visited him in Vienna, he, as an experienced scientist, gave me the following strategic advice: *“Ask critical questions provided they are based on reflections and science-based hypotheses, not on opportunistic assumptions or popular trends. Critical questions alter science.”* This formed over years my intuitive approach to develop new hypotheses aimed, in my opinion, to increase our insight into the nature of the autoimmune syndrome systemic lupus erythematosus (SLE). This idea motivated my entire scientific life—but this mantra requires humbleness: science has profound impact and consequences in all levels of the society.

There is a comprehensible and thoughtful—almost precognitive—logic link between Hans Tuppy´s “Critical questions alter science” and Oscar Wilde´s^3^ “The one duty we owe to history is to rewrite it.” History of SLE was shaped over the last 50 years^4^ through trendy assumptions and associative definitions. This narrative *is based on defense of established dogmas and (outdated? See below) conservative argumentations.* We may anticipate that knowledge in a deeper sense is—and will remain—inconsistent. This is particularly true if causally based and insightful approaches are not in focus, as recommended according to established scientific rules. From these contemplative considerations, we need to rewrite history based on science funded by contemporary, insightfully based, critical questions that are aimed to be transformed into testable hypothesis, very much as a considerate incorporation of Tuppy´s and Wilde´s mantras.

In a biological context, these mantras may lead us to scientific hypotheses based on “The causality principle” (1–3): Pathogenesis as effect of a cause. In a systemic disorder, the effect of a cause may be transformed into further downstream alternating cause-effect processes like in blood coagulation (4) or complement activation (5). This reasoning is in fact a functional definition of the causality cascade [see below (2, 6, 7)].

A problem that is somewhat difficult to comprehend is that we need *courage* to ask critical and logical questions in order to confront “the scientific establishment and its dogmas” with new paradigms and new critical hypotheses. These consequent and courageous activities materialize the duty we owe to science as an imperative to rewrite its history and to rethink and consequently revise (and even abandon)! settled historical dogmas. We have still a long way ahead to go to gain the insight necessary to understand the deeper nature of SLE and this syndrome´s delimitations (8, 9).

## Introduction

The question formulated in the title is important and contains provocative elements linked to the definition of systemic lupus erythematosus (SLE). These are of fundamental importance due to their impact in selecting and generating SLE cohorts. They are basically aimed to serve as standardized SLE study objects. Such studies aim to describe problems linked to the definition of criteria that delimitate SLE versus “SLE-like non-SLE” syndromes. Most important is to link classification criteria with two causal response qualities of criteria that are not implemented as central elements in SLE research: “the causality principle” and the consequently extended and expanding process, “the causality cascade” (see below). Both aspects inherit the theorem “cause-effect” (effect is here synonymous with clinical symptoms or criteria). Today´s science, depending on criteria-based SLE cohorts, is from a causal point of view of questionable validity. Patients are classified by criteria that are step-wise selected based on knowledge, insight, and intuition expressed by an expert panel and then elected from the selected criteria pool [i.e., Delphi panel criteria identification process (10–12)]. However, concrete studies implementing cause-effects based on the causality principle (3, 6, 13) are not visible in the main SLE classification criteria versions—although individual criteria may be discussed in this context. These versions have not collectively identified criteria directly linked to one concrete, identifiable, and unifying causal process—and maybe this is not possible?

The title of this study therefore reflects a philosophical accession to iatrogenic^5^ enigmas that adhere to our inferior understanding of the basic nature of SLE. Today, we have no clear strategy to approach sound theoretical (mechanisms/etiology) and practical (diagnostics versus classification, therapy) definitions of the syndrome (8, 14–16). The title is expressing an intellectual strategy aimed to unveil layer by layer of problems generated over decades of intense scientific activities and their results: We have to understand why we don’t understand SLE´s deeper nature! We have furthermore to dissect arguments for the idea that SLE is a syndrome with all its manifestations unified in “a one disease entity” paradigm (17)—as opposed to the SLE syndrome characterized by the non-stringent terms poly-phenotypic and poly-causal disorder(s), in other words, “SLE-like” or “SLE-like non-SLE” syndromes (18, 19). This conflictual situation can paradoxically describe SLE either as a distinct entity—”SLE as a single disease entity^6^“ or as a “group of SLE-like non-SLE disorders^7^.” Definitions in these contexts are vague and formulated on the basis of intuition and insight provided by experts in context of Delphi panels (10–12), but not on the basis of firm causality-based analyses and evidences.

SLE is today defined as a “one disease entity” (20) in the sense of Peter Hucklenbroich´s general definition of the term (21). There is, however, no consensus in this respect (3, 8). See, for example, an interesting description of 8 classified groups of 988 SLE patients in a flare study of Isenberg et al. (22). We are, pessimistically expressed, today not able to precisely delimitate the syndrome SLE from other mixed connective (SLE-like) diseases. The following discussion is problematizing our current views (whether formalized opinions or not) on SLE pathogenesis related to classification and diagnostics of SLE.

## The purpose of this reflective study is to summarize, combine, and reconcile aspects linked to the contemplative content expressed in the title of this study

This study is aimed to extend, penetrate, and combine problematic elements discussed in two previous studies (3, 8). These elements need to be concomitantly handled to find answers to problems that contribute to the definition of SLE as “an enigmatic, autoimmune syndrome.” Central is to understand if SLE is “a one disease entity” or a “poly-causal and poly-phenotypic” syndrome, in other words, if SLE is a distinctive and delimitated entity. This discussion is in accordance with sound scientific rules: SLE is an effect incited by a (distinct)? cause, whether one or more. This conflict is not problematized in relevant literature. Therefore, it is not possible from existing classification criteria versions—and their attribution rules—to construct evidences against the hypothetic statement that SLE is poly-causal in nature. There is still a strong need to recapitulate how SLE classification criteria were generated, how they were validated, and which impact they have on the composition of SLE cohorts. These problems are discussed in relevance to the title “Why is it so difficult to understand why we don’t understand SLE.” Where necessary and important in the context of a holistic discussion, basic aspects of earlier studies will be referred and re-interpreted for an attempted completion of this argumentation. This will, for the purpose of a holistic completion of this argumentation, also include dogmatic statements and problems linked to the nature of anti-dsDNA antibodies and lupus nephritis pathogenesis. This means in sum that we have to comprehend disparate dogmas that adhere to SLE: cause-effect nomination of classification criteria; definition and impact of DNA structure-specific anti-dsDNA antibodies; the inner contradiction of anti-dsDNA antibody-induced lupus nephritis.

## What is the impact of SLE classification criteria, how did they evolve over the last 50 years, and what is the theoretical definition of “a one disease entity?”

If we cannot consider the existence of a single dominant etiological factor, we cannot accordingly consider SLE as “one disease entity.” This semantic theorem will oblige us to attempt to separate, in forensic terms, “hard causal evidences” from “circumstantial indicators” in our search for the “guilty” dominant causal process(es), as discussed recently (8). To use forensic terminology for “evidence” in this context is a direct and instructive approach that can substantiate the problem: we have to search the physical footsteps of the guilty cause (*i*) to retrogradely identify the cause of effects, (*ii*) to separate SLE as “a one disease entity” from poly-etiological (and consequently poly-phenotypical) syndromes, and (*iii*) to harmonize the potentially identified causal process with new specific therapy modalities. In the context of SLE diagnostics, we have either to construct (yet by theoretical principles) homogenous SLE cohorts for detailed studies of the syndrome´s genesis*—*or we have to dismantle the current definition of the syndrome to recreate a syndrome in harmony with the causality principle: are the classification criteria causally linked to each other (as in a causality cascade), or do they reflect individual symptoms originating from individual but disparate causes? This is experimentally and analytically difficult to approach, but it may be important to give such a project a try*—*and not to remain with the pessimistic term “the enigmatic syndrome SLE.” The terms “*causality principle*” and “*causality cascade*” are essential in this context and are further discussed in detail below.

Single response elements (as criteria) adhere to and contribute to classify SLE. For example, the origin and impact of anti-dsDNA antibodies, lupus nephritis, lupus associated dermatitis, or cerebral lupus are for decades studied in detail with significant insight into their individual pathogeneses (9, 14, 15, 23, 24). A missing link in all these studies is if these central criteria are response elements linked to a common causal origin: a causality cascade. Are individual response elements encountered in SLE imposed by a common dominant cause (see e.g (14, 25–28), as exemplified by anti-dsDNA antibodies as a theoretical inducer of a causal cascade of organ manifestations (see below). These manifestations are defined as classification criteria but not as diagnostic criteria. This is a paradox that is maintained and paraphrased over decades. We encounter an intellectual conflict: anti-dsDNA antibodies are not causing SLE, and criteria are not responses to one causal origin.

It must, however, be stressed that the individual SLE classification criteria in general are not pathognomonic for SLE and consequently not diagnostic for SLE (8). In practice, clusters of criteria have been given the impact of being diagnostic since criteria clusters direct patients into SLE cohorts. These are organized as aims for studies of SLE-related scientific problems [ (3, 9); see also an insightful comment by Tsokos (29)]. This is a problematic—and a calculated paradox: SLE classification criteria classify SLE, but do not diagnose SLE—although SLE classification criteria practically endorse their impact as diagnostic criteria once a patient is enrolled into an SLE cohort!

## How was SLE basically defined as a template for SLE classification criteria, and how were they selected and fine-tuned to classify this syndrome?

It is of considerable importance to reconcile how SLE classification criteria were selected and implemented in SLE research [see, e.g (30–33), for details]. Two important problems are linked to the main SLE classification versions: it is not clear if these criteria are discussed in light of a defined or theoretical causal origin. Are they linked in a common fate destiny network based on a dominant cause, and what is the definition of SLE to which criteria were initially associated? This is a crucial point: Expert panels selected the SLE criteria based on intuition, instincts, knowledge, experience, and insight; they were subsequently statistically tested for association with SLE and the former criteria versions (31–33). This process can be formulated in the following self-confirming, but informative and provocative equation:

*A (symbolizing “criteria”) is statistically associated with B (symbolizing SLE) because B is the factor that promotes A.*

The central problem (and challenge) in this equation is *how SLE (B in the equation) was defined in context of the Delphi Panel processes (see below) performed to identify SLE classification criteria.* This intellectual problem may consequently imply that there are reasons both to trust and to mistrust the validity of current SLE classification criteria. Criteria are selected because they are instinctually believed to characterize SLE; therefore, SLE is associated with the criteria in a statistically significant way (30–34). Is this statistically supported philosophy productive, and does this philosophy reflect realities? We end up to ask: *How is indeed SLE as a template defined in this important and critical classification process—and is “the causality principle” implemented in this definition—or not?*

## The influential SLE classification criteria: versions and impact

In the following, the four main SLE classification criteria will be summarized, critically commented on, and discussed.

### The 1971 SLE preliminary classification criteria—contribution and impact

**Figure 1:**
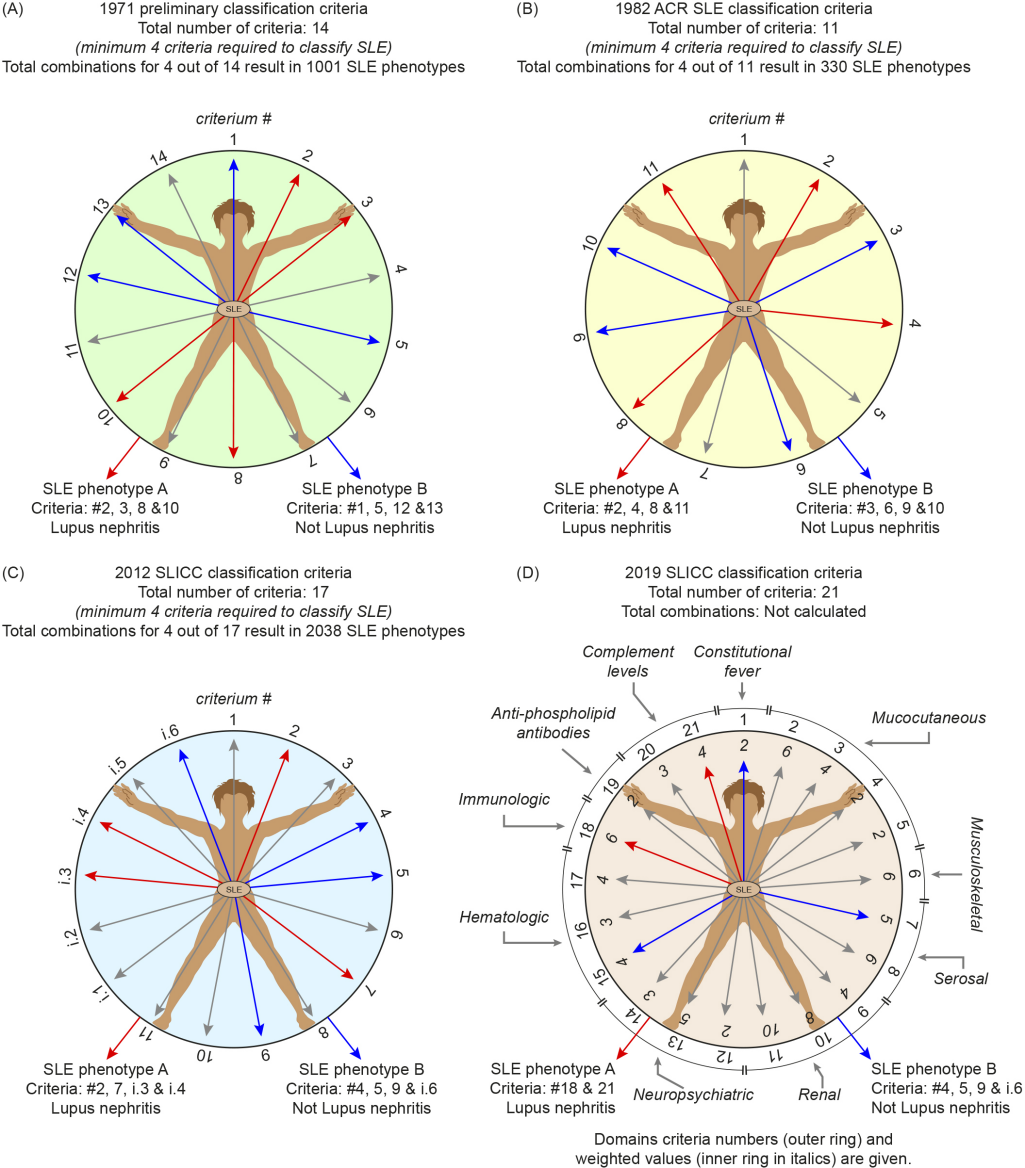
Arrangement and impact of the 1971 Preliminary, the 1982 ACR, the 2012 SLICC and the 2019 EULAR/ACR SLE classification criteria. From the first preliminary SLE classification criteria until the 2019 EULAR/ACR classification criteria, they all relate to a “body-wide” spectrum of criteria. For illustration purposes, a sketchy figure is used, inspired by Leonardo da Vinci´s The *Vitruvian Man*, the picture originally known as “*Le proporzioni del corpo umano secondo Vitruvio.”* The drawing represents Leonardo’s concept of the ideal human body proportions. The figure is useful here, and a sketch is shown for the 1971 Preliminary SLE classification criteria **(A)**, the 1982 ACR SLE classification criteria **(B)**, the 2012 SLICC SLE classification criteria **(C)**, and the 2019 EULAR/ACR SLE classification criteria **(D)**. In each Panel, two patients are demonstrated with red and blue arrows that exemplify two principally different criteria clusters. Are both suffering from SLE as “a disease entity” paradigm? Criteria that remain undetected in the patients are indicated by a grey tone. Every single criterion (as arrows) counts, but may be differently weighted in the 2019 EULAR/ACR criteria. The preliminary 1971 SLE classification criteria **(A)** incorporate 14 consensus criteria, and a minimum of 4 must be observed in a patient to classify a definite SLE. The ACR 1982 SLE classification criteria **(B)** incorporate 11 consensus criteria, and a minimum of 4 must be observed to classify a definitive SLE. The SLICC 2012 SLE classification criteria **(C)** incorporate 17 consensus criteria, and a minimum of 4 must be observed in a patient to classify a definitive SLE, and at least 1 clinical and 1 immunological criterion. An exception from this rule: A patient is said to have SLE if lupus nephritis combine with anti-dsDNA antibodies are present. The EULA/ACR 2019 SLE classification criteria **(D)** incorporate 21 weighted consensus criteria. These are organized in 10 domains. Definitive SLE classification requires at least one clinical, and one immunological criterion, and weighted criteria ≥10 points, and a positive ANA entry criterion. These criteria versions have the likely consequence that cohorts established by these criteria are poly-phenotypic (see text). For example, one patient (patient A in all panels) may be fully classified by having anti-dsDNA antibodies and renal affection (depicted by red arrows), Another patient (patient B) may be classified by presenting other criteria (depicted by blue arrows). This has the consequence that both patient A and patient B are enrolled into the same SLE cohort, and may be subjected to the same research SLE program designed to define the patient population for clinical trials and translational studies, but also to influence current understanding of the disease—with respect to etiology, pathophysiology, genetics, and responses to experimental therapies.

The 1971 preliminary classification criteria (30, 34) were selected according to Delphi panel-like praxis and statistically probed to ensure that the criteria are relevant for SLE (30). The 1971 SLE criteria version founded an authoritative rule used in later classification versions: If any 4 of the 14 criteria (numbers are valid for the 1971 preliminary classification criteria, **Figure 1A**, **Table 1**) were noticed, the patient was classified as having SLE. Consensus was reached that immunological parameters were excluded in this preliminary classification criteria version since assay principles and inter-laboratory reproducibility were not authorized at that time.

**Table 1:** Comparison* of SLE classification criteria in 4 different classification versions from 1971-2019**.

1971 preliminary SLE Classification criteria	1982 ACR SLE Classification criteria	2012 SLICC SLE Classification criteria	2019 EULAR/ACR SLE Classification criteria^#^
1.Facial erythema (butterfly rash) 2.Discoid lupus erythematosus **3. Raynaud phenomenon** **4. Alopecia** 5. **Photosensitivity** 6. Oral or nasopharyngeal ulceration 7. Arthritis without deformity **8. Lupus erythematosus cells** **9. Chronic false-positive serologic test for syphilis** 10.Profuse proteinuria 11. **Cellular casts** 12. Pleuritis or pericarditis 13. Psychosis or convulsions 14. Hemolytic anemia or leukopenia or thrombocytopenia	1. Malar rash 2. Discoid rash 3. **Photosensitivity** 4. Oral ulcers 5. Synovitis 6. Serositis 7. Neurologic manifestations 8. Renal manifestations 9. Hematologic manifestations 10. **Immunologic manifestations:** **Anti-DNA/Anti-Sm antibodies** Anti-phospholipid antibodies* 11. **ANA**	**Clinical Criteria:** 1. Acute cutaneous lupus 2. Chronic cutaneous lupus 3. Oral ulcers: palate **4. Nonscarring alopecia** **5.** Synovitis involving two or more joints or tenderness in two or more joints 6. Serositis 7. Renal disorder 8. Neurologic disorder 9. Hemolytic anemia 10. Leukopenia (< 4,000/mm3 at least once) 11. Thrombocytopenia (< 100,000/mm3) at least once **Immunological Criteria:** 1. **ANA above laboratory reference range** 2. **Anti-dsDNA above laboratory reference range** 3. **Anti-Sm** 4. Antiphospholipid antibodies* 5. **Low complement** 6. Direct Coombs test	**Obligatory Entry criterion Antinuclear antibodies** **1. Constituional fever** **2.**Acute cutaneous lupus 3.Subacute cutaneous OR Discoid lupus 4.Oral ulcers 5. **Non-scarring alopecia** 6. **Joint involvement** **7. **Pleural or pericardial effusion 8. Acute pericarditis 9. Proteinuria > 0.5g/24h 10. Renal biopsy class II OR V lupus nephritis 11. Renal biopsy class III OR IV lupus nephritis 12. Delirium 13. Seizure 14. Psychosis/delirium 15. Autoimmune hemolysis 16. Leukopenia 17. Thrombocyopenia 18. **Anti-dsDNA antibodies** 19. **Anti-Sm antibodies** 20. **Anti-Cardiolipin OR** **Anti-ß2GPI OR Lupus anticoagulant** 21. **Low C3 OR low C4 Low C3 and Low C4**

*This table demonstrates a comparison between the four major SLE classification criteria that appeared from 1971 till 2019. In this table, only criteria without comments or weighted values are given.

**Color code:

• Criterium written in brown, Raynaud phenomena, are present only in the1971 Preliminary SLE classification criteria.

• Criteria written in green are unique for the 1971 Preliminary SLE classification criteria, and the 2012 SLICC and the 2019 EULA/ACR SLE classification criteria.

• Criteria written in blue are unique for autoimmunity and inflammation and included in th 1982, 2012, and 2019 SLE classification criteria.

• Criteria written in red are shared by the 1971, 1982 criteria sets.

• Those criteria written in black are shared by all four criteria sets. Criteria may here be designated differently although they express the same. For example, “Renal manifestations, criterion # 8 in the 1982 ACR criteria, is in the 2012 SLICC criteria designated Renal, criterion # 7, and in the EULAR/ACR criteria denoted Proteinuria > 0.5g/24h (criterion # 9), Renal biopsy class II OR V lupus nephritis (criterion # 10), and Renal biopsy class III OR IV lupus nephritis (criterion # 11). These versions of criteria contain many of the same individual classification criteria and are differently annotated. These differences reflect increased insight into each criterion, and thereby different annotations, and they express the same contextual meaning.

^#^In the EULA/ACR SLE classification criteria presented in this table, only individual criteria are given. For domains, see (33).

Theoretically, random combinations of 4 of 14 criteria may result in a high number of clinical phenotypes [interestingly, this is reflected by the characterization of 8 SLE subgroups presented in an SLE flare study by Isenberg et al. (22)].

**Figure 1A** exemplifies the problem related to investigating patients assumed to suffer from a homogenous SLE syndrome. Patients are enrolled into an SLE cohort, classified by the 1971 criteria. One SLE patient in **Figure 1A** has renal manifestations (red arrows in the figure), the other patient has criteria other than nephritis (blue arrows). Are they both suffering from SLE, and do both belong to “a one disease entity?” The polyphenotypic character of SLE defined by this and other versions of SLE classification criteria reflect the impact of the classification attribution rules. Considering the polyphenotypic nature of the classified SLE, how should we then be able to develop *diagnostic criteria for SLE*? Diagnostic criteria principally relate to causality and not to the diversity of criteria selected by Delphi panel processes. Is it possible that we have created a disease that formally “cannot be diagnosed?”^8^

### Inadvertent problem

- Most probably, these criteria do not classify SLE as “a one disease entity”—rather as possible poly-etiological and poly-phenotypic “SLE-like” or “SLE-like non-SLE” syndromes. Data are missing that could explain if all—or some—of the criteria emerge from a single dominant cause in the context of, for example, “the causality cascade” paradigm.

## The 1982 ACR SLE classification criteria

**Figure 2:**
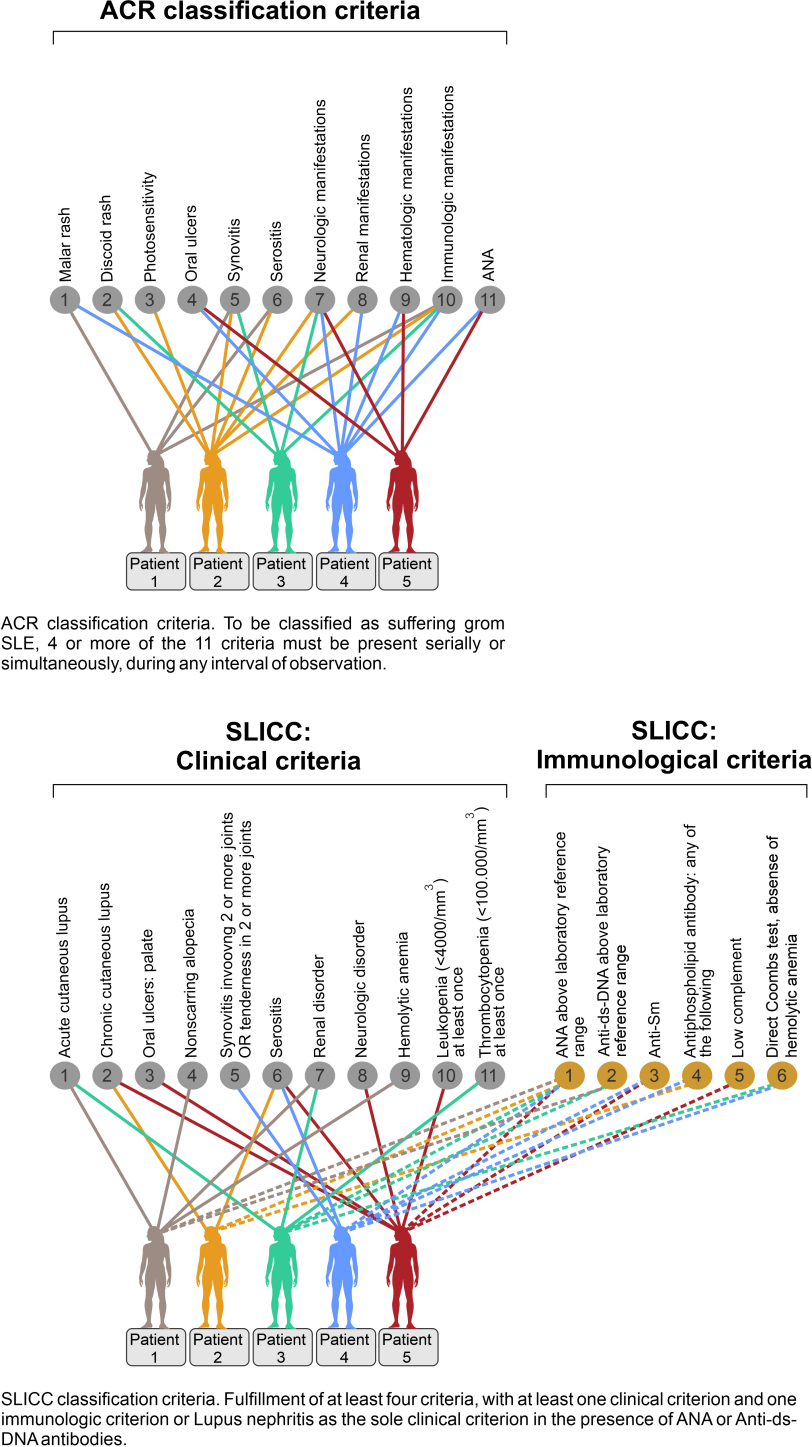
Principal problems linked to classification of systemic lupus erythematosus (SLE). Classification of SLE patients according to The ACR SLE classification criteria (upper panel), or by The SLICC SLE classification criteria (lower panel) are descriptively problematized. Each of the classification criteria systems identify a substantial diversity of clinical phenotypes. In the upper panel, five patients share some classification criteria, but diverge with respect to others, and their clinical phenotypes differ substantially. Similarly, 5 patients present different classification criteria among 11 clinical and 6 immunological SLICC criteria. Also here, the patients share some criteria, other criteria are unique to some patients. The ACR and the SLICC criteria are listed in the figure. These chaotic figures (upper and lower panels) exemplify that the use of, for example, the ACR and the SLICC criteria is problematic as basis for scientific analyses covering genetics, etiology, pathogenesis, and responses to experimental therapy in patient cohorts as the study objects do not represent a homogenous group of patients. The patients in these figures are fictive but they reflect real problems instigated by the SLE classification criteria versions. In conclusion, these criteria sets do not identify SLE as “a one disease entity” (This figure is modified from reference 35).

With the 1982 ACR SLE classification criteria, autoimmunity became implemented among the 11 selected classification criteria (31). Four of 11 criteria are required to classify SLE. It is in the article (31) stated that “*For the purpose of identifying patients in clinical studies, a person shall be said to have systemic lupus erythematosus if any 4 or more of the 11 criteria are present, serially or simultaneously, during any interval*!” This is, in fact, in contradiction to the causality principle. According to the attribution rule, the presence of autoimmunity was definitively not required to classify a patient to have SLE. **Figures 1B**, **2** (upper panel) exemplify the same problem with the 1982 SLE classification criteria as with the 1971 SLE preliminary classification criteria (**Figure 1A**, **Table 1**): one SLE patient has anti-dsDNA antibodies and lupus nephritis (red arrows), the other has other criteria (blue arrows). These alternatives challenge the dogmatic characterization of criteria-based cohorts as a homogenous “one disease entity” (see this principal problem demonstrated in **Figure 2**, upper and lower panels)! A concise question is, furthermore, if patients characterized by criteria belong to a “mono-causal” disease entity. This doubt gave birth to reservations related to investigations of SLE-related nosology, genetics, etiology, pathogenesis, and experimental therapies in SLE cohorts. These dilemmas are not convincingly problematized in the relevant central reports (11, 30–33).

### Inadvertent problems

- No evidence for or against implementation of the causality principle has been provided in the Delphi panel process [present manuscript (30–33)].
- Secondly, the attribution rule authorize “The accumulative criteria model” saying that criteria are validated if any 4 or more of the 11 criteria are present, serially or simultaneously, during any interval of observation. “ (31).

These two strategies are not harmonizing with the causality principle, and not with the Witebsky´s criteria for an autoimmune disorder (discussed below).

## The 2012 Systemic Lupus International Collaborating Clinics SLE classification criteria

The Systemic Lupus International Collaborating (SLICC) classification criteria version (32) contains 17 criteria (11 clinical and 6 immunological, **Figure 1C**, **Table 1**). The final validation was performed by comparing the SLICC criteria with the modified ACR 1997 criteria (36). The SLICC criteria performed well with higher sensitivity but lower specificity and had a lower (but still a high) number of misclassified cases [74 vs. 62 for the 1997 revised ACR criteria and the final SLICC criteria, respectively (8, 32)]. In this context: *What are the criteria used to define “misclassified,” when SLE still is regarded as an enigmatic autoimmune syndrome without clear definitions*?

The final attribution rule for the SLICC criteria authorizes that 4 criteria are required out of the 17, which gives a very high total theoretical number of criteria combinations (see **Figures 1C**, **2**, lower panel). At least one clinical and at least one immunological criterion must be identified to classify a patient as having SLE. It is stated that criteria are cumulative and need not be present concurrently (32). This disconnects the criteria from the causality principle since a potentially pathogenic anti-dsDNA antibody may appear far from being associated with, for example, lupus nephritis [discussed in (24)]. One important exception from the attribution rule is that the presence of lupus nephritis with anti-DNA antibodies is sufficient to fully classify SLE; lupus nephritis and anti-dsDNA antibodies therefore serve as *ad hoc* classification and diagnostic criteria, although the idiom “diagnostic criteria” is not a formally accepted term for SLE. An implementation of the causality principle and of the causality cascade is not visible in the 2012 SLICC criteria version (32).

### Inadvertent problems

The procedure for selection and subsequent election of classification criteria substantiate two problems that adhere to the SLICC criteria system:

- There are no reflections on whether the selected criteria are causally interrelated and interactive—in other words, they do not need to be intrinsically coherent and inter-active. The criteria may thus represent either mono-causality or poly-causality induced effects, but they may reflect responses to cause-induced causality cascade network.
- A discussion of the causality principle in context of the Witebsky`s criteria (37) for an autoimmune disease is not implemented in the 2012 SLICC SLE classification criteria (32). Individual SLE criteria are authorized even if they are detected timely unlinked from each other, similar to the two foregoing classification versions (32).

These relations may indicate that the SLICC SLE classification criteria do not define SLE as “a one disease entity,” and they are formally disconnected from the causality principle.

## The 2019 EULAR/ACR SLE classification criteria—the theoretically problematic role of the anti-nuclear antibody assay

Leuchten et al. published in 2018 a systematic literature review and meta-regression analysis of data aimed to describe the performance of anti-nuclear antibody (ANA) for classifying SLE, with the purpose to consider if ANA justified a position as a mandatory entry criterion for SLE cohorts (38). Only screening methods for ANA were emphasized. This approach minimized a discussion on which ANA sub-specificities are covered by the screening assays. Spectra of ANA specificities may differ between assay systems (39, 40). Furthermore, concise information on the diagnostic and pathogenic impact of individual ANA sub-specificities was not given attention (33, 38). For example, anti-ssDNA and anti-dsDNA antibodies (both detected in ANA assays) differ significantly in diagnostic and clinical impact [discussed in (41)]. Here, the clinicians are in conflict with the immunologists.

According to concise theoretical insight, ANA are widely detected in infections (42), cancers [(43), see a discussion in (35) and references therein], autoimmune diseases/syndromes (44), and after ingestion of certain drugs (45). *Thus, their manifestations in non-SLE contexts are substantial!* On the other hand, Choi et al. observed that 6.2% of SLE patients were ANA negative (46). It may consequently appear problematic that the 2019 EULAR/ACR SLE classification criteria recommend a positive ANA test at least once as an obligatory entry criterion for SLE classification. This is accepted, although concise ANA specificities and assay principles are not a concern. It may in this context be relevant to emphasize that the American College of Rheumatology (ACR) ANA Task Force has recommended indirect immunofluorescence on HEp-2 cells as the gold standard test for ANA detection (44), see also (40).

Selection of the EULAR/ACR SLE classification criteria was performed through an international Delphi panel exercise, and several criteria implemented in previous classification criteria versions were reiterated (see details in **Table 1**). Furthermore, data from a diagnosed patient cohort and a patient survey [see details in Aringer et al. (33)] were considered. Criteria reduction was followed by Delphi and nominal group technique discussions, while criteria definition and weighting were based on criteria performance and on results of a multi-criteria decision analysis.

The final version of the EULAR/ACR SLE classification criteria contains 21 weighted criteria organized into 10 domains (**Table 1**, **Figure 1D**). Manifestation of ANA is mandatory, and count as a criterion detected at any interval of observation. Definitive SLE classification requires at least one clinical and one immunological criterion, and weighted criteria ≥10 points, and a positive ANA entry criterion. The combination of the 21 criteria has as a clear consequence a manifold of SLE phenotypes (see a principle interpretation in **Figure 1D**, **Table 1**), as is the case for all the SLE classification criteria over the last 50 years (see details in **Figures 1A–D**, **2**).

In contrast, Schmajuk et al. opened for the view that *“*one *organ system would be sufficient for classifying SLE*” (11). This may be beneficial for investigating SLE as a syndrome dominated by, for example, lupus nephritis, and may thus represent a basis for a homogeneous SLE cohort. If this suggestion refers to affection of *any* organ system, it may be problematic and transform the cohort into being heterogeneous and less valid in SLE research contexts.

Sensitivity and specificity of the EULAR/ACR criteria compared well with the 1997 revised ACR and the 2012 SLICC SLE classification criteria. This may, however, be anticipated due to the fact that many of the 1971 criteria are reiterated in the 1982 ACR (31), the 1997 revised ACR (36), the 2012 SLICC (32), and most recently in the 2019 EULAR/ACR SLE classification criteria [(33), see **Table 1** for details]. The reiteration of criteria is *substantial*. The implementation of various clusters of weighted criteria does not, from principal reasons, support “the one disease entity “ paradigm attributed to SLE (17): The 2019 EULAR/ACR criteria may still identify SLE as a poly-etiological and poly-phenotypical syndrome (evidence for the opposite view has not been provided).

### Inadvertent problems

Problems with the 2019 EULAR/ACR SLE classification version is principally the same as for the three foregoing versions.

- The criteria are not influenced by the causality principle, as a discussion of this point is not visible in the actual reports;
- ANA (including anti-dsDNA antibodies) counts as a mandatory entry criterion if detected at least once at any interval of observation, as is stated in the 2019 EULAR/ACR SLE classification criteria (33); that is, ANA does not need to be timely linked to pathogenic conditions.

## Central problems shared between the four main versions of the SLE classification criteria

A critical problem is linked to the Delphi panel concept (11) and relies on the fact that the classified SLE patients suffer from a theoretically established and defined enigmatic disorder. SLE is in this context principally not delimitated from other “autoimmune enigmatic SLE-like” syndromes by clear definitions or by clear scientific data. There is till now no published evidence proving that the established SLE classification criteria are causally interdependent and interactive, in harmony with the causality principle and with the consequent causality cascade paradigms for complex syndromes (see below).

The SLE classification criteria were established as a systematic construction based on traditional thinking and anchored in 1971, a time with immature insight into autoimmune pathophysiology and SLE genetics. It is a striking observation that the causality principle was not debated in the 1971 preliminary and not in later classification criteria versions. Integration of the causality principle in relevant philosophical reflections anchored in scholarly principles have not been problematized over the years from 1971 to 2019, although causality has been a scientific concern over centuries, even back in antiquity (47). Thus, all the major classification criteria versions have principally the same problematic impact on causal investigations of SLE (30–33, 48). Why is it so?

Maybe we today have the knowledge sufficient to comprehend that the modern definition of SLE implies that SLE is not a single disease entity. *Do we simply lack the courage to split the syndrome into different causal-related versions?* These could be characterized as contrasts to the less distinct and enigmatic “overlapping lupus-like” disorders [discussed in (19)]. SLE may remain enigmatic until we investigate the syndrome in a complicated and cumbersome way in light of the causality principle, with the implementation of important and highly relevant central historical data (see anti-dsDNA antibodies and lupus nephritis discussed below). These data have been revitalized recently (3, 8, 41, 49). The causality principle may be the single sine qua non distinction that may enable us to intuitively and scientifically designate diagnostic criteria and to sub-fractionate the diagnosis of SLE by different unique cause-effect relations. If the causality principle is included as an understatement during the development of classification criteria, this should have been specified and precisely described.

We have to strive to define the distinction between “SLE” and “SLE-like non-SLE” diseases. *There is no unifying conceptual quality expressed by clustered classification criteria that can provide, advance, and practically inform about the integrated complex mechanistic nature of SLE*. Exceptions are given for some rare, well-described gene deficiencies or deviations (50–57). Such gene deficiencies are principally divided into two groups, one caused by a single gene (monogenic) defect, the other by polygenic defects. The monogenic SLE version is easier to understand—a single gene defect forms the basis for one basic pathophysiological aberration that in turn may promote downstream alternating cause-effects. In other words, monogenic SLE is a potential example of induction of a causality cascade where symptoms (or effects or criteria) are offspring of the original monogenic defect (53–57). Wild-type SLE—or “SLE-like non-SLE”—syndromes are not comparatively easy to comprehend. This group of disorders may rely on polygenic defects and an interplay between the genetic and environmental factors. This is a more complex picture and is still difficult to understand and to delimitate. A simple consequence will be that classification criteria promoted by a single gene defect truly belong to a causality cascade and are synchronized, interactive, and interdependent, while classification criteria promoted by polygenetic and environmental factors may appear as criteria clusters timely unlinked from each other, have poly-causal origins, and do not reflect effects consistent with the causality cascade paradigm.

Monogenic SLE and “wild-type” SLE conditions may be valid models to compare repertoires of early and late SLE classification criteria appearing in the two separate conditions. Such studies are still awaited [critically discussed in (3, 8, 49)], and may have a strong impact on new therapeutic modalities like the implementation of chimeric antigen receptor T cells (CAR T cells).

## Chimeric antigen receptor T cell therapy for autoimmune diseases including SLE: Is CAR T cell therapy promising in poly-phenotypic and poly-causal SLE cohorts—and why?

Wild-type SLE in this context may be a synonym for poly-phenotypic (and possibly poly-causal) SLE. How does this comply with SLE cohorts established by criteria that may be unlinked and not appear as interactive, as opposed to models involved in the causality cascade paradigm? Based on this dilemma, we may question if such cohorts are useful as study objects to investigate if, for example, genetics, etiology, pathogenesis and experimental therapy modalities comply with “a one disease entity” paradigm [discussed in (3, 8, 9)].

There are, however, pathogenic situations where new promising therapeutic modalities have been described (58, 59). Therapies developed using classification criteria for patient inclusion have been outstandingly successful for the chimeric antigen receptor T (CAR T) cellular therapy. The premises for successful treatment of polyphenotypic and polycausal SLE variants characterized by autoimmune organ manifestations are if immune tolerance can be restored. T cells that are modified to express chimeric antigen receptors on T cells (CAR T cells) have been investigated. CAR T cells target B cells to selectively deplete or to down-regulate autoimmune responses responsible for systemic or organ-specific autoimmune disorders (discussed in e.g (58, 59). In this scenario, CAR T cells may be effective in patients with various autoimmune B cell activities (58) or in refractory SLE (59); that is, whether they produce a single pathogenic organ-specific autoantibody or multiple autoimmune antibody specificities. This is a very promising therapeutic progression and may work well irrespective of whether patients suffer from mono- or poly-causal SLE variants. Future research efforts should, from this observation, analyze if CAR T-cell therapies are successful in genuine SLE (like monogenic or monocausal SLE) as well as in disparate autoimmune SLE-like non-SLE situations.

## Do SLE classification criteria help us to understand SLE? Disregarded historical data and paradigms have reduced our contemporary SLE-related insight and concise definitions

There are many reasons to assume why classification criteria may reduce our understanding of the nature of SLE. A steadily increasing number and clusters of SLE classification criteria will not provide us with more science-based insight into the core process(es) that promote and maintain SLE.

A central question in this context needs profound considerations: What is the impact, in biological terms, of concrete and disparate criteria clusters (defined as minimum required number of criteria) in different SLE patients? Do different clusters reflect the same etiology-based pathogenic process? Is it possible to solve this problem when we must consider that the criteria count irrespective whether they appear simultaneously or one by one uncoupled in time [the accumulative criteria model (31–33)], and more problematic, do different criteria clusters irrespective of their composition provide us with information applicable to quite different SLE phenotypes? In other words, do different criteria clusters reflect a common cause?

An idealized reflection: SLE classification criteria may appear coincidently as disparately composed clusters that represent consequences of a single cause. Criteria clusters as mirror images of a causality cascade may reflect a downstream alternating cause-effect network introduced by a primary single cause. If so, we have to understand why one cause may promote quite different criteria clusters that involve different organ manifestations from patient to patient. Is this realistic scenery? Or do we have to re-think our strategies for selection of cause-related criteria?

Are then all these clusters ultimately unique parts of “genuine” SLE? This critical question opens for the intriguing possibility that SLE indeed is representing “a one disease entity” despite its polyphenotypical nature. To solve this eventuality, it is needed to perform, for example, retrograde investigations to search the causal origin of individual criteria and the origin of the inciting cause(s).

## Can consequences of single gene defects help us to understand origin of poly-phenotypic SLE?

**Figure 3:**
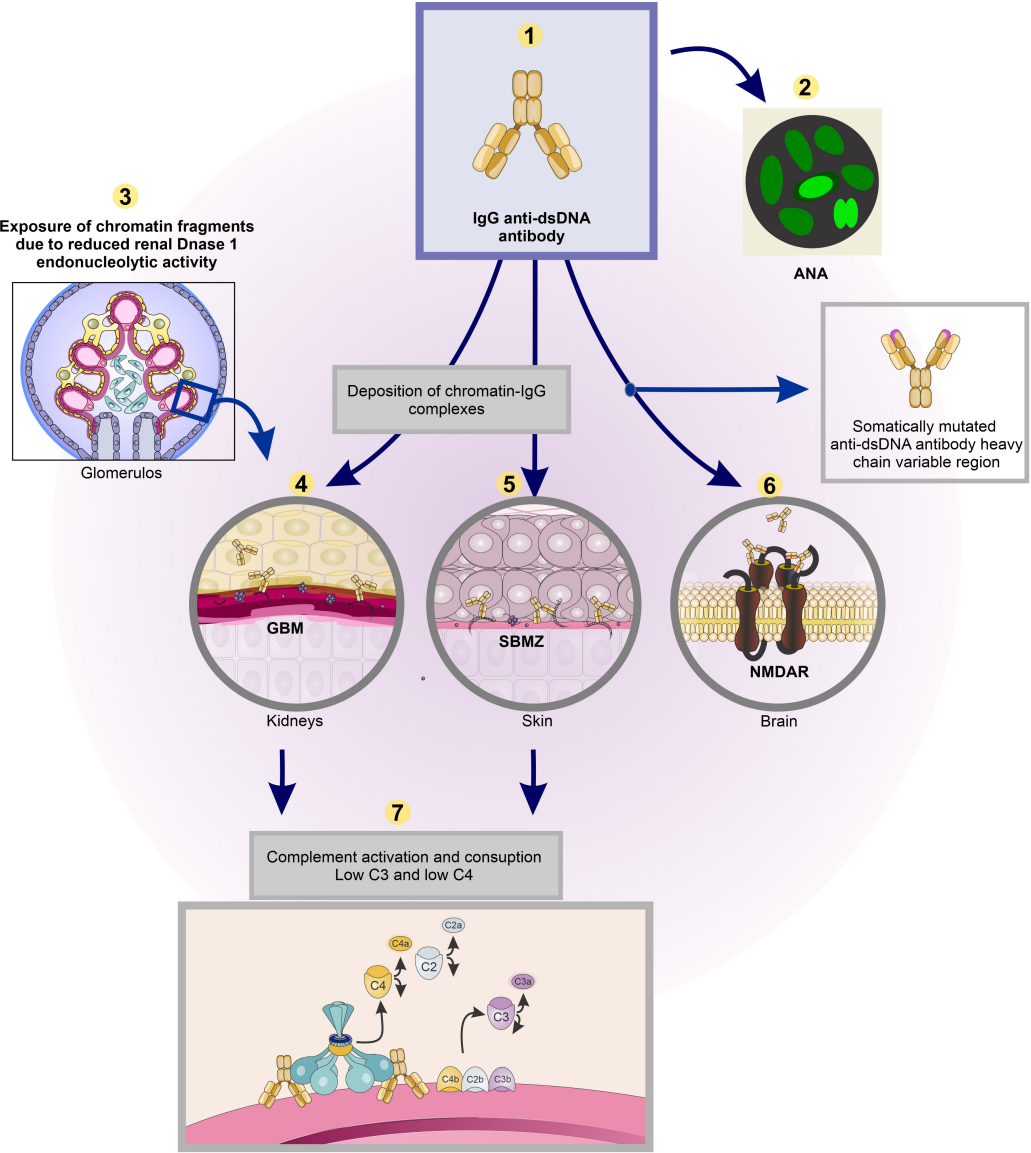
Identification of an exemplified central and interactive set of criteria to categorize SLE as an alternative to classify SLE by classification criteria—the causality cascade aspect. The clinical criteria linked to anti-dsDNA antibodies are lupus nephritis (4), lupus dermatitis (5), and cerebral lupus (6). As an inciting factor, termination of tolerance to dsDNA result in affinity-mutated anti-dsDNA antibodies. These antibodies may promote a causal and consequent activation of an interactive network of pathophysiological events that account for the following SLE-associated measures: (1) anti-dsDNA antibodies, (2) ANA, (3) exposure of chromatin secondary to anti-dsDNA mediated silenced renal DNase 1 endonucleolytic activity (see text for details), (4) lupus nephritis, (5) lupus dermatitis, (6) lupus brain disease incited by cross-reaction of somatically mutated anti-dsDNA antibodies with NMDAR, and finally (7) complement activation and consumption— low C3 and low C4. *At least fulfillment of criteria number 1–3 in combination with any of criteria 4–7 is equivalent to a classified and diagnosed SLE syndrome.* Except for silencing of renal DNase 1 gene (here criterion #3), all these criteria are, and have been, influential authorities as diagnostic criteria, as they are pathophysiologically involved in SLE. *Criterion #3, exposed chromatin fragments, is a central partner that transform anti-dsDNA from being a clinical epiphenomenon into a significant pathogenic factor (see text for details)*! Thus, the 7 criteria are interrelated and interactive basically due to termination of tolerance to dsDNA (and to exposure of chromatin constituents). This is a sine qua non for appearance of the criteria listed in the figure: They serve as cardinal symptoms in SLE. The figure is a solid example of a causality cascade (see text). This Figure has been previously used in an open access journal (3). ANA, antinuclear antibodies; MM, mesangial matrix; GBM, glomerulus basement membranes; SBMZ, skin basement membrane zone; NMDAR, *N*-methyl-D-aspartate receptor (also known as the NMDA receptor - NMDAR).

In line with these reflections, an association of oligo- or poly-phenotypic SLE with a single gene defect may promote the idea that the interrelated criteria are timely synchronized in contrast to what would be likely by chance alone. Secondly, a monogenic origin of SLE is expected to result in syndromes with a lower degree of criteria cluster variability than variability observed in “wild-type” SLE [discussed in (55)]. Is it possible to define differences between monogenic SLE and SLE promoted by an interplay of disparate causes? A metaphor, SLE disposes for infections (60–64), and infections may directly promote, for example, dsDNA/chromatin autoimmunity (65–71), which in turn may cause subsequent inflammatory effects (as criteria), consistent with the causality cascade paradigm (see an example of a causality cascade in the theoretical **Figure 3**). A confusing question is whether infection promotes an additional cause for SLE or if infection is caused by an underlying SLE-disposing cause. We still encounter fundamental problems inherited in the SLE classification criteria versions that make SLE difficult to understand, irrespective whether of mono- or polygenic or other origins.

A similar problem is relevant for ANA, a term that implements unspecified anti-nucleosome/anti-chromatin autoimmunity. ANA is required as an entry criterion in the 2019 EULA/ACR SLE classification criteria and is relevant in any SLE or SLE-like conditions, which all may be difficult to delimitate toward each other. These autoantibodies are frequently detected as drug-induced non-pathogenic, infection-induced, or cancer-associated transient or sustained autoimmunity [(27, 43, 45, 71), discussed in (27)]. It may be problematic that unspecified ANA is weighted as a mandatory entry SLE classification criterion. But, if present, it may add to the autoimmune character of SLE in a patient.

## Summarizing these considerations, the following collection of reflective and critical questions needs to be answered

- Is SLE a monocausal-driven disease?
   o Do the plethora of formally accepted criteria reflect one causal (mono-etiological) origin? If not,
   o is the idea of SLE as “a disease entity” compatible with a “poly-etiological”/multi-causal SLE?
- What are the concerns, arguments, and intentions related to the concrete rules responsible for the evolution of the current SLE criteria selections (30–33)?
   o Intuition and insight?
   o Logic based on experience?
   o Delphi panel reduction processes—again based on “insight and experience?”
   o Are Delphi panel opinions simply based on firm scientific data or on terms like “impressions,” “experience with patients,” “traditions,” “insight/expertise,” or “instinctual processes.” ^9^
- Is the causality principle implemented in Delphi panel discussions and decisions that convincingly demonstrate influence on current classification criteria versions? If so, where in the relevant literature can we read and learn from such concise reflections?
- Finally, how is the implementation and impact of the diversity of criteria into a holistic syndrome SLE basically explained? What is a strict etiology-based definition of the SLE syndrome in this context?

Depending on the answers to these questions, we need to generate new radical hypotheses that reflect implementation of the causality principle (1, 3, 13) to understand, to diagnose, and to treat SLE. If we disregard this approach, we will return to the “*pre-Robert Koch and Louis Pasteur era*,” an historical era characterized by studying symptoms and “intuitive, philosophy-based therapy modalities,” without indication of causal hypotheses (72). The Koch-Pasteur era is characterized by a radical paradigm shift in medical science, and is the starting point where studies of symptoms transformed into studies of their causes (72)! This opened for causal therapy principles and for vaccination to protect against causative contagious infections (72). From the current state of understanding, we need a new open-minded discussion and new critical hypotheses based on the Koch-Pasteur derived causality principles to explain and treat SLE.

## The basic causality principle and the causality cascade: Complex mechanisms in a complex syndrome

The causality principle (2, 6) is the most basic principle we have to consider in our search for a phenotypic time-kinetic (or cause-effect) definition of SLE [discussed in (3, 49)]. The causality principle in its simple version declares the relation between a cause and its effect (response element). The principle has impact on several levels relevant to SLE research (73).

- Equal effects results from equal causes;
- Distinct causes have distinct, reiterated and recognizable effects in complex scenarios;
- The causality cascade proclaims that one cause can initiate subsequent response elements that in turn transform into alternating downstream causes and effects (6, 7, 13, 73, 74). One cause can impose a selection of effects as in complex syndromes like SLE. Theoretically, criteria groups may tentatively be analysed in a retrograde approach step by step to identify the initial phenomenological (etiological) cause – if this is possible (see a model of a causality cascade in **Figure 3**).

Examples of causality cascades that are operational in

 o Coagulation (4)

 o Complement activation (5)

 o Anti-dsDNA antibody induced complex causality cascade [(3), see theoretical example in **Figure 3**].

The principal causality cascade model is not reported in the central classification criteria publications [see, e.g (17, 30–33, 36, 48, 75, 76)]. In these publications, the Delphi panel approach (10, 11) has taken over for the functional causality approach. The Delphi panel approach has by its instinctual nature hindered causality from being the theoretically most central element in SLE classification processes.

This pessimistic view is further underscored if we observe that there is no radical paradigm shifts if we study and compare the four major SLE classification criteria versions with each other; many of the criteria are reiterated (**Table 1**), and attribution rules are principally common for all of them.

The paradigm “one cause - one effect” may at first glance be irrelevant for the complexity of the SLE syndrome. There is actually one interpretable solution to this complex situation. If we, for example, study the clinical involvement of anti-dsDNA antibodies as a functional cause, we may be able to trace different apparently incongruent response elements primarily and secondarily imposed by the anti-dsDNA antibodies. As outlined in **Figure 3**, the anti-dsDNA antibody—as a causal factor—promotes central downstream criteria with high impact in SLE [for discussions, see (3, 27, 35) and references therein]. These central criteria, incited by one causal factor, practically exemplify a causality cascade. “Causal structure” is the relevant term to be used here (7).

This paradigm of a mono-causal, poly-phenotypic SLE is in analogy with, for example, the causality cascade that characterizes a poly-phenotypic syndrome incited by SARS-CoV-2; the long COVID-19 syndrome. Therefore, the COVID-19 is regarded as “the new great imitator.” (77)^10^. In a clinical context, this syndrome is poly-phenotypic, but basically mono-causal, as the virus is the sole initial causal factor. The virus as the primary disease-promoting factor may, in certain situation, initiate a cascade of downstream response elements that account for the polyphenotypic character of the long COVID-19 syndrome.

## If we search to understand what we do not understand about SLE, a discussion on clinical impact of DNA structures and anti-dsDNA antibodies must be integrated in this complicated and holistic discussion

“The anti-dsDNA antibody” possesses characteristics that comply with the problem proclaimed in the title of this study. Anti-dsDNA antibodies are enigmatic, origin not conclusively defined, specificities for DNA structures have been disregarded, and their role as diagnostic marker inconclusive. The anti-dsDNA antibody holds an archaic position as a central classification, diagnostic and pathogenic factor in SLE. For decades, the antibody had a strong influence on SLE classification, diagnostics, and autoimmune pathogenesis. These aspects have been substantially modified in recent years. Despite this strong focus, historically important information on DNA structures targeted by highly specific antibodies have been neglected—even in today´s modern studies on SLE.

This part of the study concentrates on three unique and central issues, communicated here as questions: (*i*) Does an antibody specific for dsDNA unequivocally reflect the diagnosis SLE and the pathogenic process(es) in SLE? (*ii*) Does the antibody separate SLE from “SLE-like non-SLE syndromes?” (*iii*) Does “the anti-dsDNA antibody” represent one specificity, or disparate DNA structure-specific antibodies? These are fundamental questions we are not able to answer today because relevant scientific data have been neglected in the history of immunogenic DNA structures and SLE [discussed in (41)].

There are three problematic, unresolved, and poorly investigated aspects that adhere to anti-DNA antibodies: (*i*) their distinctive structural DNA specificities (41); (*ii*) Their clinical impact (27); and (*iii*) What do the antibodies recognize in kidneys in context of lupus nephritis—exposed chromatin fragments or inherent cross-reactive basement membrane structures [discussed in detail in (24)].

Conflicting scientific data on the nephritogenic effect and modus operandi of anti-dsDNA antibodies have been available over decades but hardly implemented in appropriate scientific protocols (24, 27). Still, relevant historical data are either neglected or regarded as being of low importance in central scientific studies.

Today, the anti-dsDNA antibody is regarded as a unique biomarker for SLE and has reached a position as an unquestionable immunological dogma [discussed in e.g (25, 27, 28)]. There are strong reasons to critically revise the impact of this dogma from a strict scientific point of view (41). In fact, inadequate integration of classical knowledge and central historical data may explain why we still adhere the term *“enigmatic autoimmune syndrome”* to SLE (9, 14, 15, 17, 23, 78). This is discussed below point by point.

### Anti-DNA antibodies were first described in bacterial infections in 1938–1939—about 20 years before their detection in SLE

Recent years` literature on anti-dsDNA antibodies notoriously state that these autoantibodies were first described in SLE in 1957 [(79–82), see an account in (27)]. This has established a problematic dogma stating that these antibodies adhere to SLE as an essential biomarker. What is true, however, is that anti-dsDNA antibodies are not pathognomonic for SLE (27), but they may be pathogenic in SLE, as they may account, for example, lupus nephritis, dermatitis, and cerebral lupus (24, 83–86). The pathogenic impact of anti-dsDNA antibodies depends on the accumulation of large, undigested extracellular chromatin fragments in, for example, GBM as a consequence of SLE-related loss of the renal endonuclease DNase 1 (discussed in (24), see below). It has been overlooked that these antibodies were detected during bacterial infections already in the late 1930s (87–89), as is also more recently described in viral infections [see, e.g., (69–71, 90, 91)]. If the 1938–1939 reports were recapitulated in the aftermath of the 1957 observations, research on SLE-related autoimmunity would probably have taken other directions based on firmer hypotheses. This has unfortunately resulted in problematic interpretative conflicts in SLE research—conflicts that are still active.

### Anti-dsDNA antibodies: impact and conflicts—historical data versus contemporary trends and dogmas

Anti-dsDNA antibodies are not unique biomarkers for SLE! Already in 1957, scientists did not seriously consider that these antibodies could be driven by bacterial infections (87–89). These publications may have been neglected because they are methodologically demanding to read! This fact may explain why bacterially infected patient groups were not or only rarely implemented in population studies aimed to determine the clinical impact of these autoantibodies. We must now seriously consider if anti-dsDNA antibodies maintain their pathogenic potentials when linked to bacterial and viral infections (27, 91–94). Infection-derived antibodies are produced according to the hapten-carrier systems [viral origin (71, 90, 91, 95–97)] and to cross-stimulation by immunogenic CpG rich bacterial DNA (92–94). These mechanistic models have been ascribed active and specific production of the antibodies *in vivo*. SLE-related and SLE-independent bacterial and viral infections (70, 98–101) may hence account for anti-dsDNA and anti-chromatin antibodies. They are consequently not unique for SLE. The unawareness of this fact has overestimated their diagnostic impact in SLE (25, 27, 28).

Thus, anti-dsDNA antibodies are not unreservedly linked to SLE. The strange reason for this is that the 1938/1939 studies have only seldom been cited in relevant DNA autoimmunity contexts (27). Infection-derived origin of anti-dsDNA antibodies has during recent years experienced a renaissance during the central studies of Gilkeson and Pisetsky. They clearly demonstrated the mechanism for induction of anti-dsDNA antibodies by CpG-rich bacterial DNA (92–94, 102, 103). These observations add to the many tentatively proposed mechanisms for induction of anti-dsDNA antibodies. We have still no firm ideas that could explain which mechanisms are responsible for spontaneous DNA/chromatin autoimmunity in, for example, SLE. genetics, reduced clearance of apoptotic secondary necrotic chromatin, NETs, hormones, infections, or external influence (104–110)? Conclusive data are still awaited (27, 41). If we can increase our insight into these problems, this may be of significant importance when we search to understand autoimmunity in SLE and to solve one aspect of why it is difficult to understand why we don’t understand SLE.

Since bacterial (62, 98, 101, 111, 112) and viral (63, 70, 99, 113–115) infections are obligate in SLE (60–64), one may hypothesize that productive viral and bacterial infections may promote immune stimulation resulting in anti-dsDNA/anti-chromatin autoimmunity. This would most probably involve the hapten-carrier mechanism in accordance with the Sercarz hapten-carrier theorem (116, 117), as is experimentally described and confirmed (71, 90, 118). An alternative would be active cross-stimulation by bacterial CpG-rich DNA-protein carrier complexes (92–94). These infectious-based models may therefore explain one pathway resulting in anti-dsDNA antibody production *in vivo*.

If we focus on infectious-induced anti-dsDNA antibodies, this may lead us into concrete investigations (25, 71, 91, 97, 101, 119) aimed at identifying the origin of DNA-specific autoimmune responses—also in SLE. Considering the referred data in the 1938/1939 reports and the neglection of the manifold of well-known immunogenic DNA structures, it may be wise to cite Ludwik Fleck^11^ (120) on the impact of history on the present status of knowledge and its consequences:

*“For the current state of knowledge remains vague when history is not considered, just as history remains vague without substantive knowledge of the current state.”*

In order to increase our understanding of SLE, we must critically reconsider the scientific impact of historical data; here, related to anti-DNA antibodies. Such old data are highly relevant in our search for problem solutions.

### Anti-DNA antibody assays—principles and DNA structure targets: The incomprehensible term “The anti-dsDNA antibody” is irrational in clinical studies

The statement saying that anti-dsDNA antibodies play a significant role as a classification criterion and a diagnostic factor for SLE remains by definition as inconsistent dogmas, according to central data on DNA structures [reviewed in (41)]. This critical notion is manifest if the molecular structure of the target DNA and the corresponding assay principles are not defined and implemented in antibody assay strategies (41, 121, 122).

For example, DNA is not only “ssDNA” or “dsDNA” as referred to in the relevant literature. DNA presents a manifold of gene expression-associated function-related structures, such as ssDNA, elongated B dsDNA, bent B dsDNA, Z DNA, cruciform DNA; and viral DNA, bacterial DNA, synthetic DNA derivatives, and other dynamic structures reflecting the status of DNA functions [**Table 2** (135–139), discussed in (41)]. These constructs are not “frozen, nonflexible, and dead” structures; they are central dynamically changing—still immunogenic—elements that are intimately linked to DNA functions within the genetic machinery (138, 140).

**Table 2:** Dynamically changing DNA structures are immunogenic but clinical specificities of induced antibodies are largely not investigated.

DNAstruc-ture	Immunogenic?	Ref	Autoimmunogenic?	Clinical specificity	REF	Comments	REF
B DNA elongated	Yes#	(65, 90, 123)	Yes	SLE* Infections, cancer	(9, 27)	inconsistently investigated	(41)
B DNA Bent		(97, 123)	Yes	SLE* Others?	(124, 125)	inconsistently investigated	(41)
ssDNA		(122, 126)	Yes	Clinically unspecific	(41)	Even detected in healthy individuals	-
Cruciform DNA	Yes	(127)	Not determined	Not examined	N/A	Unknown clinical specificity	(41)
Z DNA	Yes	(128, 129)	Yes	SLE-other	(130, 131)	Needs more clinical studies	(41)
Viral DNA**	Yes	(70, 91, 132)	Not actual per definition	Autoimmunity in viral infections	(71, 90, 91, 95–97)	May induce for example, anti-dsDNA antibodies by hapten-carrier mechanism*	(8, 71, 116)
Bacterial DNA	Yes	(92, 94)	Not actual per definition	Autoimmunity in bacterial inf.	(87, 89, 92–94, 102, 103)	May induce autoantibodies	(41, 92, 133, 134)

*Depends on control groups; **Refers to polyomavirus DNA; ^#^Elongated B DNA is immunogenic provided it is in complex with an immunogenic carrier protein.

Without insight into these DNA structures and their critical impact in corresponding quantitative and qualitative anti-DNA antibody assays, these assay results are principally uninterpretable, because we do not consider which DNA structures are recognized in which assay principle, and with which clinical situation each specific antibody correlates with (27, 28, 41). These diverse DNA structures and their individual and specific functions have been published over several decades related to genetics and biochemistry, but are in the autoimmune and SLE-linked scientific studies and contexts not considered important (41).

The different DNA structures inherit different immunogenic potentials (see information in **Table 2**). This may open for different clinical impacts of the various anti-DNA structure- specific antibodies [see, e.g., early preliminary discussions by David Stollar (141–143)]. Notably, in this context, anti-dsDNA structure-specific antibodies are not defined or commented on in the current SLE classification criteria publications.

**Figure 4:**
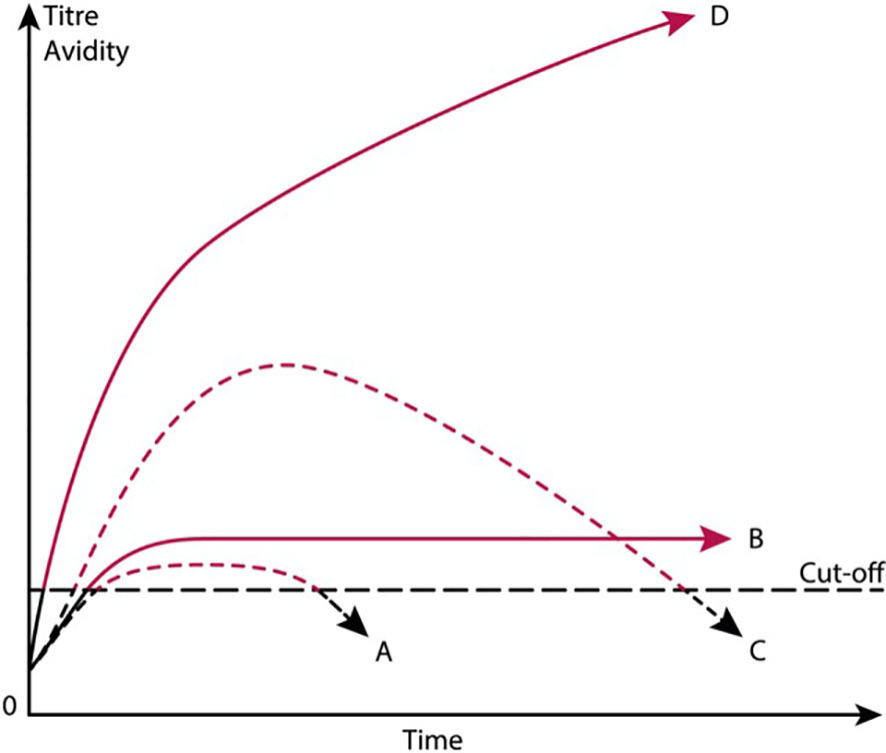
Theoretical anti-dsDNA antibody profiles in context of systemic lupus erythematosus (SLE) classification criteria. The 1982 ACR, 2012 SLICC SLE, and the 2019 EULAR/ACR SLE classification criteria versions include anti-dsDNA antibodies as a criterion. As a criterion the antibodies are poorly and unsubstantially defined and explained. Are all anti-dsDNA antibodies linked to SLE, and are they all pathogenic? For example, as demonstrated in the figure, a short-lived stimulus by an infectious agent may induce transient anti-dsDNA antibodies at low titers **(A)** (91, 95, 134). If the infectious stimulus persists, the anti-dsDNA antibody may continue at low titers, even though *above the assay cutoff level* **(B)**. The anti-dsDNA antibody production in **(C)** is transient, although at high titers, as a consequence of a strong, transient stimulus either of autologous or, for example, infectious origin. In **(D)**, the immune response is characterized by sustained production of anti-dsDNA antibodies at high to very high titers. The red parts of each profile represent autoantibody levels above the antibody cutoff levels as defined by ACR or SLICC criteria. To this quantitative problem, it should be added that the term “dsDNA” is not defined and specified. Today, the “dsDNA term” is covered by bent or elongated B DNA, Z DNA, cruciform DNA, and even bacterial and viral dsDNA [see a detailed discussion in the text and in (41)]. The curves are fictive and constructed empirically in order to demonstrate the variability of anti-dsDNA antibody profiles, all of which fulfill requirements in the SLE classification criteria for SLE. This figure was first published in (9).

As an extension of these principle problems, the current SLE classification criteria require certain limits for anti-dsDNA antibodies to count as a definitive criterion; for example, above a certain analytical cut off value. This is inconsistent and not sufficient to implement a given antibody as an SLE classification. Antibody profiles in clinical medicine may vary, from high and stable titers, or high but transient titers, to low and transient profiles (see theoretical profiles in **Figure 4**). Every profile illustrated in **Figure 4** fulfills the requirements defined in the SLE classification criteria [discussed in (8)]. This problem reduces or even detriments our understanding of anti-dsDNA antibodies as classification, diagnostic, and pathogenic factors.

### ANA—fluorescent patterns and diagnostic interpretations

Another problem deals with the clinical impact of fluorescent ANA patterns in a diagnostic context. “The ANA interpretation criteria” published by the International Consensus on ANA Patterns, have been reported to recognize ANA patterns with different clinical relevance - or the absence thereof—considering the more than 30 patterns (44). Only some of these patterns are associated with SLE. This is problematic from two basic arguments. For the first, an ANA is often produced in context of other anti-chromatin antibodies or other ANAs (27, 28). These combinations will have impact on fluorescent ANA patterns. Secondly, hardly any ANA (with a possible exception for anti-Sm antibodies) provides us with strong diagnostic impacts (27, 28). Even anti-dsDNA antibodies are not specific for SLE (see text for details).

### According to classification rules, ANA count irrespective when they appear, and their link to clinical situations are formally not required

This attribution rule, as defined in the SLE classification criteria versions, should be reconsidered—they are not harmonizing with the Witebsky´s (37) Koch-derived (144, 145) criteria that characterize an autoimmune and an infectious disease. Clearly, unlinking anti-dsDNA autoantibodies timely from concise clinical symptoms unlinks the autoantibody from the causality principle (32, 33). This is a conflictual problem, and disregard a concise understanding of their pathogenic impact. This problem is also indirectly described in a study by Arbuckle et al. (146). In that study, anti-dsDNA antibodies could be detected years ahead of overt SLE. The role of these antibodies in the pre-SLE period is unclear, but it is demonstrated that the pure presence of anti-dsDNA antibodies does not necessarily predict corresponding autoimmune inflammatory organ affections. The anti-dsDNA autoantibodies require an accessible antigen as a partner to initiate inflammatory processes, like extracellularly exposed chromatin fragments, evidently due to an acquired error of chromatin metabolism (i.e., loss of renal DNase 1 mRNA and enzyme activity, discussed in (24, 35, 41), and below).

### Pathogenesis of Lupus nephritis: Do anti-dsDNA antibodies recognize chromatin fragments exposed in glomerular basement membranes, or do they bind inherent glomerular basement membrane structures?

The pathogenic mechanism(s) accounting for lupus nephritis is not conclusively resolved. Two antibody-dependent nephritis models explain the descriptive theories. In one model, the antibodies are pathogenic because they cross-react with inherent glomerular membrane/matrix structures like laminin or entactin (discussed in (3, 24, 49), equivalent with an immune-mediated Type 2 inflammation (147). An alternative model declares that anti-dsDNA antibodies bind exposed GBM-associated chromatin fragments (148–150). This model inherits a cooperative two-folded process; progressive loss of renal DNase 1 endonuclease account for extra-cellular accumulation of large undigested chromatin fragments in GBM where they are targeted by anti-dsDNA/anti-chromatin antibodies (151–153). This will appear as a process complying with a Type 3 immune complex mediated inflammation (147).

The latter model, “the chromatin exposure model” harmonizes with a dynamic progressive loss of renal DNase 1 enzyme activity (151–153). The DNase 1 deficiency is strongly linked to a progressive nephritic process from mesangial nephritis (chromatin-IgG deposits in mesangium) into end-stage kidney disease. Undigested chromatin fragments in mesangial matrix and in GBM, therefore, serve as renal neo-targets for anti-dsDNA and anti-chromatin autoantibodies (24). These two conflicting models have been known for decades, but few attempts have been performed to validate if one or both models are really operational *in vivo*.

These conflicting models are in the literature reluctantly discussed with no clear conclusions (154–158). The understanding of the nephritic process is a prerequisite to develop relevant causal therapy modalities for lupus nephritis, as the two models must theoretically be treated differently, thus linking causal modes with causal therapy modalities [discussed in (159–161)].

The central paradigm unequivocally declares that loss of renal DNase 1 enzyme activity is a critical event that renders anti-dsDNA antibodies nephritogenic (24). This may give preference for the chromatin model as dominant. To definitively conclude, further penetrating investigations are needed to describe the role of both models and the real nature of lupus nephritis ( (24, 148–150).

## Concluding remarks

### Anti-dsDNA antibodies: their individual DNA structure specificities related to diagnostic and pathogenic impacts

- Anti-dsDNA antibodies were described in bacterial infections 18–19 years before these antibodies were described in SLE. DNA-specific antibodies are still, however, paradoxically characterized as unique, archetypical markers for SLE. This viewpoint must be reinvestigated and probed based on hypotheses focusing on relevant data on origin of anti-dsDNA antibodies and their specificities for DNA structures.
- DNA structure specificities and assay principles in clinical contexts are still not given concern in classification criteria versions (30–33). This leaves these biomarkers incomprehensible (41, 138) and contributes to the enigmatic character of SLE.
- As discussed elsewhere, different assay principles detect strictly different antibody specificities against disparate DNA structures. Notably, these distinct anti-DNA antibody specificities have nothing to do with inherent and individual antibody avidities, as has been claimed (27, 124, 162, 163).
- The immunogenic potential of each of these DNA structures differ, and specific antibodies may correlate with different clinical affections, and some few may even be produced in normal individuals [see **Table 2** (141–143)]. This problem needs further investigations in light of historical and recent data on DNA structures (3, 41).
- According to rules defined in the recent classification criteria versions, anti-dsDNA antibodies count irrespective if they even appear timely unlinked from any clinical situation.
- From this argumentation, there is an imperative need to analyze the diagnostic and pathogenic impacts exerted by the individual DNA structure-specific antibodies.

### General conclusive comments

Adequate integration of classical insight and central historical data, relevant to pathogenic processes in SLE, is a major element in this study. We need to prioritize to understand why we don’t understand the pathobiology having effects on SLE as an assumed delimitated unit, and to determine what we mean with the complicated term: SLE as “a one disease entity,” aside from the term: “SLE as an enigmatic autoimmune syndrome.”

In forthcoming studies on classification and diagnostic criteria, it is essential to implement “the causality principle” and the consequent downstream events covered by the term “the causality cascade” in new versions of Delphi panel-like processes; causality must be a central element. Causality reflections may principally open for new cause-related therapeutic prototype principles (159, 164–166), discussed in (24).

The consequent purpose of this contemplative theoretical and fact-based study is to answer the question expressed in the title of this study: *“Why is it so difficult to understand why we don’t understand SLE”*—the SLE syndrome that indeed is still embraced by the term “the enigmatic autoimmune syndrome SLE.”

## Data Availability

The original contributions presented in the study are included in the article/supplementary material. Further inquiries can be directed to the corresponding author.

## Footnotes

2 Professor Hans Tuppy was born 22 July 1924. He graduated in Chemistry from the University of Vienna in 1948. After graduation he went to work in the world-famous laboratory of Fred Sanger on the sequencing of insulin. His time in Cambridge followed by research work in the Carlsberg Laboratory in Copenhagen, from where he returned to the University of Vienna. Later in his career, he shaped Austria’s scientific landscape as Minister of Science and as Rector of the University of Vienna among many other positions. *Copied from: Max Perutz Labs.*

3 Oscar Wilde (born 16 October 1854, Dublin, Ireland—died 30 November 1900, Paris, France) was an Irish wit, poet, and dramatist whose reputation rests on his only novel, The Picture of Dorian Gray (1891), and on his comic masterpieces Lady Windermere’s Fan (1892) and The Importance of Being Earnest (1895). He was a spokesman for the late 19th century Aesthetic movement in England, which advocated art for art’s sake. *Britannica*.

4 For example, SLE classification criteria, instituted in 1971, are not based on hard, concrete and measurable scientific cause-effect analyses. The problematic ignorance of clear consequences of cause-effect integration has reduced the impact of classification and diagnostics of SLE—as is discussed in this study.

5 Iatrogenic: Something induced unintentionally by a physician or surgeon or by medical treatment or diagnostic procedures.

6 “SLE as a single disease entity” is a constructed term since SLE is, with few monogenic exceptions, a syndrome with poorly defined etiological origin(s) and with poly-phenotypical clinical courses.

7 “SLE-like non-SLE syndromes” is also a constructed term, similar to the contrary term “SLE as a single disease entity.” It is important to separate the two terms from each other and to comprehend their semantic and logic impacts. This is clear due to absence of cause-effect (or diagnostic) definitions implemented in the classification criteria.

8 I once heard the following statement from a central SLE-oriented scientist: “You can´t define SLE, but you know what it is when you see it.”

9 The Delphi panel program is a systematic process of forecasting using the collective opinion of panel members. The structured method of developing consensus among panel members using Delphi methodology has gained acceptance in diverse fields of medicine. The Delphi methods assumed a pivotal role in the last few decades to develop best practice guidance *using collective intelligence where research is limited, ethically/logistically difficult and evidences are missing* (10).

10
https://www.yalemedicine.org/conditions/long-covid-post-covid-conditions-pcc

11 Ludwik Fleck (1896–1961) was a Polish physician, biologist and philosopher. In the 1930s he developed the philosophical concepts of "Denkstil" ("thought style") and the "Denkkollektiv" ("thought collective"). Fleck´s concept of the "thought collective" is central in the philosophy of science and in logology (the "science of science") and was a leading force in the Polish school of logology. He used his concept to describe nonlinear evolution of scientific ideas in a “thought collective,” much as in Thomas Kuhn´s "periodic paradigm shift" concept (9).

## In memoriam

This study is dedicated the memory of Professor Hans Tuppy, who died 24 April 2024. He was my first mentor. The systems science presented in this study is influenced by years of problematic considerations, discussions, curiosities, and by attempts to understand why we do not fully understand pathobiological processes characteristic of the enigmatic syndrome SLE. This curiosity was instigated by Hans Tuppy during and in the aftermath of my medical study in Vienna. In this context, I also want to acknowledge central international scientists for vivid discussions over decades. They tried to explain what I still don’t understand: Is SLE “a one disease entity” or “a group of SLE-like non-SLE syndromes.” They have formed my intuitive understanding of SLE and the role of SLE-associated autoimmunity. Their influence on my considerations have toned down some of my more provocative and radical theories.
